# Supplementary material for: Splice-Junction-Based Mapping of Alternative Isoforms in the Human Proteome
Source: Cell Rep. Author manuscript; Available in PMC 2020 Jan 15. (PMC6961840; doi:10.1016/j.celrep.2019.11.026)
Supplement: 2 [file NIHMS1546469-supplement-2.zip › DF1/PXD010154/Liver/Liver_20_CC2D1B_GMNLPAPPGVTPDDLDAFVRFEFHYPNSGGPQAR.pdf]

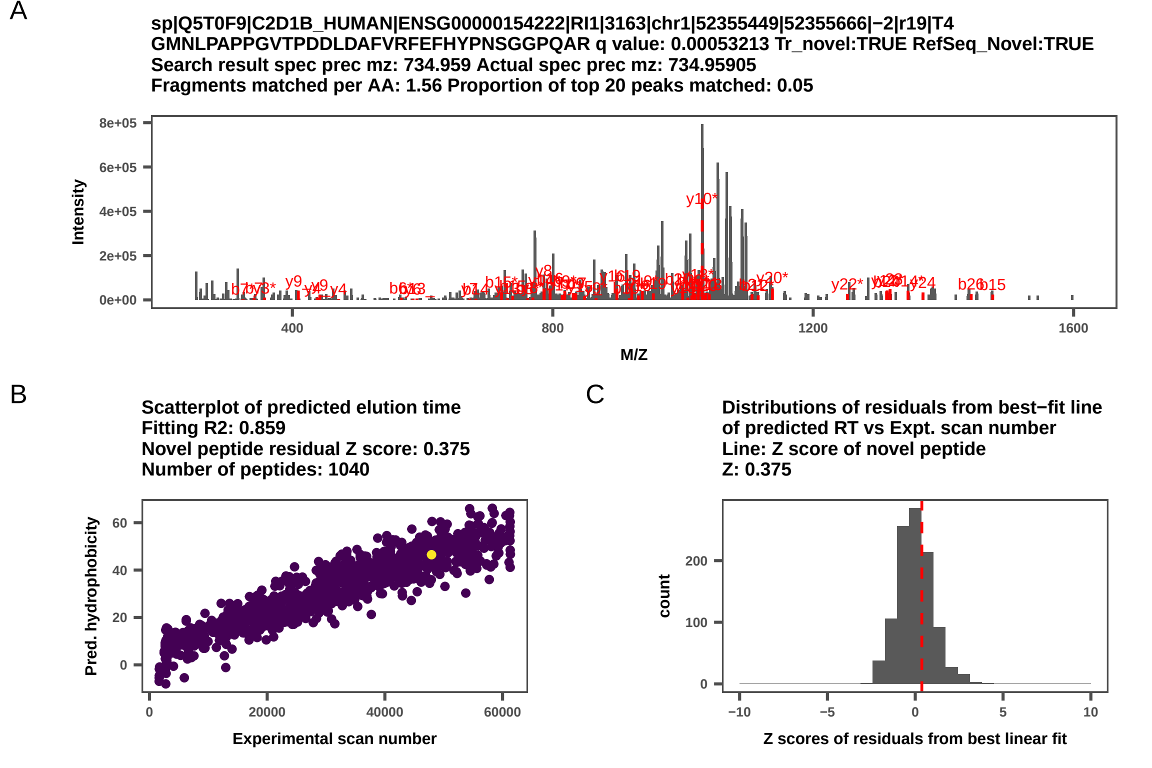
A
sp|Q5T0F9|C2D1B_HUMAN|ENSG00000154222|RI1|3163|chr1|52355449|52355666|−2|r19|T4
GMNLPAPPGVTPDDLDAFVRFEFHYPNSGGPQAR q value: 0.00053213 Tr_novel:TRUE RefSeq_Novel:TRUE
Search result spec prec mz: 734.959 Actual spec prec mz: 734.95905
Fragments matched per AA: 1.56 Proportion of top 20 peaks matched: 0.05
Intensity
8e+05
6e+05
4e+05
2e+05
0e+00
y10*
400
800
1200
1600
M/Z
B
Scatterplot of predicted elution time
Fitting R2: 0.859
Novel peptide residual Z score: 0.375
Number of peptides: 1040
Pred. hydrophobicity
60
40
20
0
0
20000
40000
60000
Experimental scan number
C
Distributions of residuals from best−fit line
of predicted RT vs Expt. scan number
Line: Z score of novel peptide
Z: 0.375
count
200
100
0
−10
−5
0
5
10
Z scores of residuals from best linear fit
